# The Spatiotemporal Distribution and Drivers of Urban Carbon Emission Efficiency: The Role of Technological Innovation

**DOI:** 10.3390/ijerph19159111

**Published:** 2022-07-26

**Authors:** Ruijing Zheng, Yu Cheng, Haimeng Liu, Wei Chen, Xiaodong Chen, Yaping Wang

**Affiliations:** 1 College of Geography and Environment, Shandong Normal University, Jinan 250358, China; zrjbeibei@163.com (R.Z.); weichen@sdnu.edu.cn (W.C.); 2 Institute of Geographic Sciences and Natural Resources Research, Chinese Academy of Sciences, Beijing 100101, China; liuhm@igsnrr.ac.cn; 3 College of Management, Sichuan Anticultural University, Chengdu 611130, China; chenxiaodongtzxy@gmail.com

**Keywords:** carbon emission efficiency, super-SBM model, spatiotemporal pattern, technological innovation, panel data models, urban agglomerations

## Abstract

Urban agglomerations have become the core areas for carbon reduction in China since they account for around 75% of its total emissions. Beijing-Tianjin-Hebei (BTH), Yangtze River Delta (YRD), and the Pearl River Delta (PRD), which are its most important poles of regional development and technological innovation, are key to achieving China’s carbon peak emissions target. Based on the panel data of these three major urban agglomerations from 2003 to 2017, this study estimated the carbon emission efficiency (CEE) by the super-efficiency slacks-based measure (super-SBM) model and analyzed its spatiotemporal distribution pattern. The Dagum Gini coefficient was used to evaluate the difference in CEE between the three major agglomerations, while panel data models were established to analyze the impact of technological innovation on the three agglomerations. The overall CEE showed an upward trend during the study period, with significant spatial and temporal variations. Additionally, the main source of urban agglomeration difference in CEE evolved from inter-regional net differences to intensity of transvariation. While technological innovations are expected to significantly improve CEE, their effect varies among urban agglomerations. These results provide policymakers with insights on the collaborative planning of urban agglomerations and the low-carbon economy.

## 1. Introduction

Climate change is widely recognized as a grave threat to humanity [1,2]. The increase of greenhouse gases caused by anthropogenic activities such as the consumption of fossil fuels, deforestation, fertilization, and industrial processes has led to climate change, especially global warming [3]. Recent years have shown increasing temperatures and a high frequency of extreme weather events in various regions [4], which threaten the survival and development of mankind [5]. Therefore, mitigating global warming is a common responsibility of both developed and developing countries.

China has entered a key stage of transition from a low-income to a middle-income country through the process of industrialization, which requires a large amount of energy [6]. Due to the long-term dependence on the high input, high emission, and low output of its development patterns, large consumption of resources is inevitably behind the growth of economic prosperity, which hinders the high-quality development and green transformation of China’s economy. China surpassed the US as the largest carbon emitter in 2007 [7] and accounted for around 30% of the world’s CO_2_ emissions in 2019. To seek a sustainable development pattern and deal with climate change, the Chinese government actively undertakes the responsibility of emission reduction and formulates emission reduction plans. At the Paris Climate conference in 2015, the Chinese government proposed to decrease the country’s carbon intensity by 60–65% by 2030 compared to 2005. Moreover, at the 75th Session of the UN, the Chinese government officially declared that it would reach its carbon emissions peak by 2030 and carbon neutrality before 2060. This goal puts forward higher requirements for improving energy-savings and carbon emissions.

Driven by the new normal and the new urbanization development strategy in China, the construction and development of urban agglomerations have gradually been elevated to an unprecedented height. In particular, Beijing-Tianjin-Hebei (BTH), the Yangtze River Delta (YRD), and the Pearl River Delta (PRD), the three major urban agglomerations located on the eastern coast of China, are regarded as important growth poles for promoting regional economic growth. In 2018, these three economic zones accounted for 43.48% of the national GDP, with 28.41% of the total population [8]. However, the three major urban agglomerations are also responsible for China’s largest energy consumption and most conspicuous environmental pollution [9]. Therefore, the contradiction between high resource consumption, carbon emissions, and economic development of the three major urban agglomerations needs to be urgently resolved.

Carbon emission efficiency (CEE) is an essential indicator for measuring green and sustainable development, whose improvement can help achieve a “win-win” between carbon reduction and economic transition. Since innovations constantly change the external environment people depend on [10], technological innovation has become a fundamental variable in low-carbon development and sustainability performance enhancement. Technological innovation is one of the essential tools to improve energy productivity and it reduces energy costs to a certain extent [11]. It can be divided into two categories: green technological innovation and non-green technological innovation. Green technological innovation is the new outcomes of pro-environmental innovations including carbon capture technology and low-carbon technology, which can simultaneously reduce emissions and improve energy efficiency. In contrast, non-green technology innovation focuses on generating economic benefits by increasing productivity, which ignores improving environmental quality to some extent. Since CEE measures the relationship between carbon emission and economic development from the perspective of input and output, the impact of technological innovation on CEE is the result of the trade-off between the two types of innovation. Although CEE and its driving factors in China have been discussed extensively at the provincial [12,13] and the sectoral levels [14,15], there are still uncertainties regarding the effects of technological innovation on urban CEE. Theoretically, technological innovation can achieve clean production and reduce energy consumption through equipment renewal, which promotes the high-polluting industries’ transition to low-carbon sustainability. Conversely, enterprises may ignore environmental protection in the process of pursuing technological innovation to increase production efficiency and economic value, which will produce more carbon dioxide. This study attempts to analyze the CEE of China’s three major urban agglomerations from a comparative perspective to answer the following four questions: (1) Are the spatiotemporal distribution patterns of CEE of the three major urban agglomerations consistent? (2) Where does their unbalance come from? (3) What roles do different technological innovation indicators play in CEE? (4) Are there obvious differences in the impact of technological innovation on different urban agglomerations? To explore these questions, this study proceeded as follows. First, a total factor-based framework was proposed to measure CEE using the super-efficiency slacks-based measure (super-SBM) model considered with undesired output and comprehensively analyzed the spatiotemporal distribution pattern in the three major urban agglomerations. Second, the Dagum Gini coefficient was introduced to estimate CEE differences in the three major urban agglomerations, which better reveals the composition and source of spatial disequilibrium of CEE. Third, the influencing drivers of technological innovation on CEE and their heterogeneous impact on different urban agglomerations were identified by panel data models. Practical strategies are provided to enhance the CEE of each urban agglomeration adapting to local conditions.

In response to the shortcomings of previous studies, this paper aims to make a contribution in two ways: (1) enrich the research framework of urban CEE by considering regional differences from the perspective of urban agglomerations and (2) further clarify the impact of different technological innovation indicators on CEE and their spatial heterogeneity. The remaining content of this study is structured as follows: Section 2 provides a review of the existing literature, Section 3 describes the research methods and selection of indicators, Section 4 presents the empirical results, Section 5 presents conclusions and the last section provides some policy implications.

## 2. Literature Review

Despite CEE having attracted much attention in recent years, there is still no clear and unified definition in academia. From the single-factor perspective, Sun (2005) argued that using CO_2_ emissions per unit of GDP to measure CEE is an important criterion for evaluating a country’s energy conservation and emission reduction [16]. Mielnik and Goldemberg (1999) proposed the concept of a carbon index, which is defined as the carbon emissions per unit of energy consumption [17]. Other single-factor indicators, such as carbon intensity [18] and carbon productivity [19], have also been widely regarded as measures of CEE. However, these definitions only consider the proportion of carbon emissions to GDP (or energy consumption) and ignore the multi-dimensional input factors in the actual production process. Recently, most studies estimated CEE from the total factor perspective [20,21]. Common input–output methods include data envelopment analysis (DEA) and stochastic frontier analysis (SFA). For instance, Yu and Zhang (2021) adapted a non-convex meta-frontier DEA model to calculate CEE from 251 Chinese cities over the period of 2003 to 2018 [22]. Jiang et al. (2020) used the super-SBM model and Malmquist index to evaluate the CEE of the logistics industry from a transportation strategy perspective [23]. This typical input–output relationship can measure CEE more comprehensively.

To further understand CEE changes at different times in different regions, many scholars have focused on their spatiotemporal patterns and evolution characteristics. For example, Wei et al. (2019) applied non-parametric kernel density estimation to analyze the dynamic evolution of CEE in 97 countries and found that the changes in distribution were relatively stable during the study period [24]. Moreover, spatial autocorrelation analysis [25], spatial Markov probability transfer matrix [26], Theil index [27], and K-means clustering [28] have also been extensively used in exploring the spatial-temporal pattern of CEE. However, existing studies analyzed the temporal and spatial distribution of CEE in China, mainly concentrating on provincial and regional scales. Moreover, there is still a huge research gap regarding urban agglomerations. Especially given the rapid rise of China’s urban agglomeration, relevant research needs to be urgently carried out at this scale. Sources of spatiotemporal differences also need to be recognized.

The drivers of CEE have been passionately discussed [29,30]. In the study of Quan et al. (2020), factors such as economic output, energy consumption structure, government intervention, and population size are inversely proportional to CEE [31]. Liu et al. (2018) analyzed the influencing process on urban agglomeration’s CEE from four effects of urbanization and found that different effects significantly impact on CEE in different ways [32]. However, existing studies show that the correlation between technological innovation and carbon emissions is controversial. One view states that green technological innovation can reduce CO_2_ emissions in the shorter term, further strengthening environmental sustainability [33]. Wang et al. (2019) pointed out that technological progress is the main driver to promote CEE [34]. Another view is that technological innovation increases carbon emissions, while it will reduce pollution and carbon emissions with economic development [35]. Additionally, the relationship between technological innovation and CEE is heterogeneous in different regions and sectors. Li and Cheng (2020) measured the total factor CEE of 31 manufacturing industries in China from 2012 to 2016 and found significant differences across technology-intensive industries [36]. Regression models are widely used for exploring driving mechanisms, such as the spatial regression model [37], the Tobit model [38], and the Global Vector Autoregressive approach [39]. Although there has been a lot of research on the correlation between technology and CEE, the jury is still out on how technological innovation influences CEE from the perspective of different urban agglomerations.

## 3. Methodology

### 3.1. Study Area

**Figure 1:**
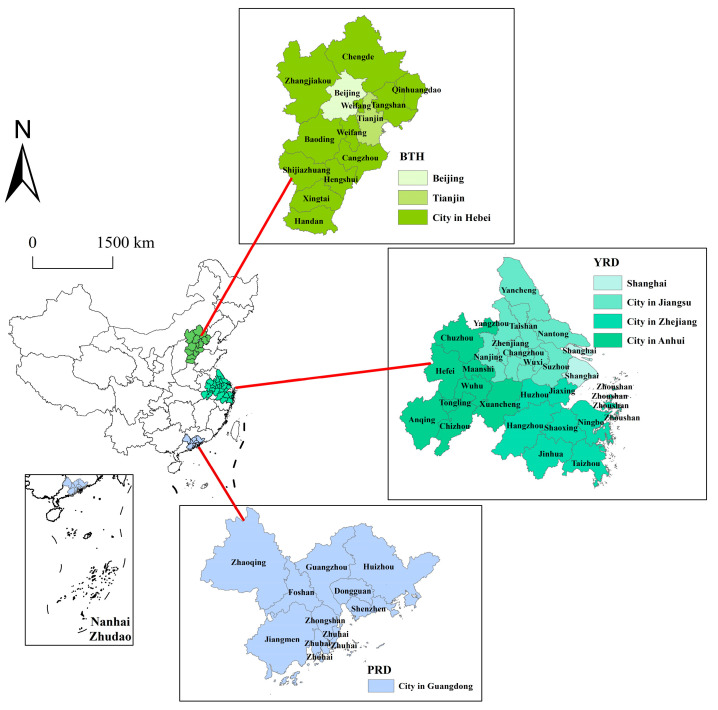
The location of each city in the three major urban agglomerations.

As urban agglomeration has become the dominant mode of new-type urbanization construction in China, the BTH, YRD, and PRD are regarded as the core regions for promoting the high-quality development of China’s economy. Geographically, the three major urban agglomerations are located on the most bustling east coast of China. The BTH plays a crucial role in the Bohai Economic Rim and Northeast Asia, which includes 2 municipalities, Beijing and Tianjin, as well as 11 cities in Hebei province. In 2019, its GDP reached about 8.46 trillion yuan, accounting for 8.5% of the national total. The YRD is situated on the middle and lower reaches of the Yangtze River, forming a vibrant cluster with Shanghai as the economic center, which consists of 26 cities (i.e., Shanghai, Nanjing, Wuxi, Hangzhou, Ningbo, Hefei, etc.). The PRD possesses flourishing manufacturing and foreign trade, including nine cities in Guangdong province. In 2018, the urbanization rate of the PRD reached 85.3%, which is the highest among China’s urban agglomerations. This study selected the three major urban agglomerations as the research area and the panel data were collected from 2003 to 2017 (Figure 1).

### 3.2. Super-SBM Model

The DEA method, firstly proposed by Charnes et al. (1978), evaluates the efficiency of decision-making units (DMUs) with multiple inputs and outputs [40]. However, the traditional DEA model ignores the measurement error caused by slack variables. To overcome this limitation, Tone (2001) introduced the non-radial and non-oriented slack-based measure (SBM) model [41]. However, the efficiency value of DMUs is often equal to 1 in both the traditional DEA and SBM models, which fails to compare and distinguish effective DMUs. Andersen and Petersen (1993) proposed a super-efficiency DEA model which allows effective DMU values greater than 1 [42]. Tone (2002) further defined the super-SBM mode, combining the SBM and super-DEA model advantages, and the SBM model was modified to consider undesirable outputs in the production process [43]. The super-SBM model deals with undesirable outputs and compares each DMU efficiency value, which is widely used to measure total factor productivity, ecological efficiency, energy efficiency, etc. This study used the super-SBM model with undesirable outputs to estimate the CEE at the city level. The model involves the following steps:

First, assume that a production system has *n* DMUs, and each DMU utilizes *m* inputs (*x*) to create *S*_1_ desirable outputs (*y^a^*) and *S*_2_ undesirable outputs (*y^b^*). Those three vectors are defined as: $x\in R^{m}$, $y^{a}\in R^{S_{1}}$, and $y^{b}\in R^{S_{2}}$. The matrix of *X*, *Y^a^*, and *Y^b^* can be expressed as follows:

(1) $$X=\left[x_{1},x_{2}\cdots x_{n}\right]\in R^{m\times n}$$

(2) $$Y^{a}=\left[y_{1}^{a},y_{2}^{a}\cdots y_{n}^{a}\right]\in R^{S_{1}\times n}$$

(3) $$Y^{b}=\left[y_{1}^{b},y_{2}^{b}\cdots y_{n}^{b}\right]\in R^{S_{2}\times n}$$

Presuming X>0, $Y^{a}>0$ and $Y^{b}>0$, the production possibility set (*P*) is defined as follows:

(4) $$P=\left\{\left(x,y^{a},y^{b}\right)|x\geq \lambda X,y^{a}\leq \lambda Y^{a},y^{b}\geq \lambda Y^{b},\lambda \geq 0\right\}$$

where P indicates that the actual desirable outputs are lower than the frontier desirable outputs, and the actual undesirable outputs are higher than the frontier undesirable outputs.

Second, based on the production possibility set, the SBM with undesirable outputs can be formed as follows:

(5) $$\rho =\min\frac{1-\frac{1}{m}\sum_{i=1}^{m}\frac{S_{i}^{-}}{x_{ik}}}{1+\frac{1}{S_{1}+S_{2}}\left(\sum_{r=1}^{S_{1}}\frac{S_{r}^{a}}{y_{rk}^{a}}+\sum_{r=1}^{S_{2}}\frac{S_{r}^{b}}{y_{rk}^{b}}\right)},s.t.\left\{ \begin{array}{l} x_{k}=\lambda X+S^{-}\\ y_{k}^{a}=\lambda Y^{a}-S^{a}\\ y_{k}^{b}=\lambda Y^{b}+S^{b}\\ S^{-}\geq 0,S^{a}\geq 0,S^{b}\geq 0,\theta \geq 0 \end{array}\right.$$

where ρ stands for objective efficiency in the rage [0,1], $S=\left(S^{-},S^{a},S^{b}\right)$ denotes the slack variables of inputs, desirable outputs, and undesirable outputs, respectively, and λ is the weighted vector. Only when ρ=1 (i.e., $S^{-}=S^{a}=S^{b}=0$) can the specific DMU_k_ ($x_{k},y_{k}^{a},y_{k}^{b}$) be efficient. Otherwise, if 0≤ρ<1, the evaluation of DMU_k_ is inefficient in the SBM model, which needs to be improved in inputs and outputs. To calculate more reasonable efficiency values, the corresponding super-SBM model can be written as follows:

(6) $$\rho^{*}=\min\frac{\frac{1}{m}\sum_{i=1}^{m}\frac{{\bar{x}}_{i}}{x_{ik}}}{\frac{1}{S_{1}+S_{2}}\left(\sum_{r=1}^{S_{1}}\frac{{\bar{y}}_{r}^{a}}{y_{rk}^{a}}+\sum_{r=1}^{S_{2}}\frac{{\bar{y}}_{r}^{b}}{y_{rk}^{b}}\right)},s.t.\left\{ \begin{array}{l} \bar{x}\geq \sum_{j=1,\neq k}^{n}\lambda_{j}x_{j}\\ {\bar{y}}^{a}\leq \sum_{j=1,\neq k}^{n}\lambda_{j}y_{j}^{a}\\ {\bar{y}}^{b}\geq \sum_{j=1,\neq k}^{n}\lambda_{j}y_{j}^{b}\\ \bar{x}\geq x_{k},{\bar{y}}^{d}\leq y_{k}^{d},{\bar{y}}^{u}\geq y_{k}^{u},\lambda_{j}>0 \end{array}\right.$$

where the value of $\rho^{*}$ can be greater than 1, which effectively deals with the problem of ranking the SBM-efficient DMUs.

According to previous studies [44,45], three perspectives of capital inputs, labor input, and energy input are considered into the system of the CEE input–output index. Fully considering the data collection and availability, fixed assets investment, number of employees, and electricity consumption are selected as the input indicators. As the fixed capital stock cannot be directly obtained, this study adopted the approach of Liu et al. (2016) for calculation [46]. Based on the study of Zhang and Liu (2022) [47], the GDP and carbon emissions of each city were selected as the desired output and undesirable output, respectively. The CEE input–output indexes are listed in Table 1.

### 3.3. Dagum Gini Coefficient

The traditional Gini coefficient is a statistical measure of economic inequality, also commonly used to estimate the geographic distribution of inequality. The Dagum Gini coefficient decomposes the regional differences into three parts, effectively identifying the source of spatial disequilibrium. Based on the research by Dagum (1997) [48] and Han et al. (2020) [49], the Dagum Gini coefficient is defined as:

(7) $$G=\frac{\sum_{j=1}^{K}\sum_{f=1}^{K}\sum_{i=1}^{n_{j}}\sum_{h=1}^{n_{f}}\left|y_{ji}-y_{fh}\right|}{2n^{2}\mu }$$

where *G* denotes the total Gini coefficient, which stands for the total difference of CEE between cities in China. *K* is the number of urban agglomerations, including BHT, YRD, and PRD, and j=1, 2,⋯, K; f=1, 2,⋯, K. μ is the mean value of all cities and *n* is the number of cities in the delineated urban agglomeration. $y_{ji}$ and $y_{fh}$ are the CEE of cities in the *j*-th and the *f*-th urban agglomerations, respectively. Before decomposition of the total Gini coefficient, the average CEE of each urban agglomeration is ranked first, $\bar{Y_{f}}\leq \cdots \bar{Y_{j}}\leq \cdots \leq \bar{Y_{k}}$.

The Dagum Gini coefficient can be decomposed into three contributions: intra-regional differences ($G_{w}$), inter-regional net differences ($G_{nb}$), and intensity of transvariation ($G_{t}$). The relationship among contributions satisfies the following formula:

(8) $$G=G_{w}+G_{nb}+G_{t}$$

Each part is calculated as follows:

(9) $$G_{w}=\sum_{j=1}^{K}G_{jj}p_{j}s_{j}$$

(10) $$G_{jj}=\frac{\sum_{i=1}^{n_{j}}\sum_{r=1}^{n_{j}}\left|y_{ji}-y_{fh}\right|}{2{\bar{Y}}_{j}n_{j}^{2}}$$

(11) $$G_{jh}=\frac{\sum_{i=1}^{n_{j}}\sum_{r=1}^{n_{f}}\left|y_{ji}-y_{fr}\right|}{n_{j}n_{f}({\bar{Y}}_{j}{\bar{Y}}_{f})}$$

(12) $$G_{nb}=\sum_{j=2}^{K}\sum_{f=1}^{j-1}G_{jf}(p_{j}s_{f}+p_{f}s_{j})D_{jf}$$

(13) $$G_{t}=\sum_{j=2}^{K}\sum_{f=1}^{j-1}G_{jf}(p_{j}s_{f}+p_{f}s_{j})(1-D_{jf})$$

Equation (10) measures the Gini coefficient of the *j-*th urban agglomeration, which denotes the distribution difference of CEE among cities in an urban agglomeration. Equation (11) measures the Gini coefficient between the *j*-th and *f*-th urban agglomerations. In Formulas (12) and (13), $G_{jf}$ stands for the distribution difference between urban agglomeration to the total Gini coefficient. $p_{j}=n_{j}/n$, $s_{j}=n_{j}\bar{Y_{j}}/\left(n\bar{Y}\right)$, j=1, 2, ⋯,K. $D_{jf}$ is the relative impact of the CEE between the *j*-th and *f*-th urban agglomerations, and the specific formulas are:

(14) $$D_{jf}=\frac{d_{jf}-p_{jf}}{d_{jf}+p_{jf}}$$

(15) $$d_{jf}=\int_{0}^{\infty }dF_{j}(y)\int_{0}^{y}\left(y-x)dF_{f}(x\right)$$

(16) $$p_{jf}=\int_{0}^{\infty }dF_{f}(y)\int_{0}^{y}\left(y-x)dF_{j}(x\right)$$

where $d_{jf}$ is the *D*-value of the CEE between the *j*-th and *f*-th urban agglomerations, which can also be interpreted as the weighted average of all $y_{ji}-y_{fh}>0$ samples in the *j*-th and *f*-th urban agglomerations. $p_{jf}$ represents the first moment of transvariation, which is interpreted as the weighted average of all $y_{fh}-y_{ji}>0$ samples in the *j*-th and *f*-th urban agglomerations. $F_{j}\left(F_{f}\right)$ is the cumulative density distribution function. According to the above formulas, we measured and decomposed the Dagum Gini coefficients of the distribution difference of CEE among the three urban agglomerations.

### 3.4. Variables’ Explanation

The purpose of this study was to explore how technological innovation affects the CEE at the city level. Except for the technological innovation level, this study preliminarily focused on the driving factors of the CEE from four perspectives: urbanization level, industrial structure, economic development level, and foreign trade. We also selected six secondary indicators that may affect the CEE to quantitatively analyze the driving mechanism (Table 2).

(1) Technological innovation level: Technological innovation plays an important role in saving energy and enhancing CEE. According to the study by Xie et al. (2021) [50], technological progress reduces carbon emissions and facilitates energy-saving by urging the industrial sector to improve production methods. It also promotes the efficiency of production and economy. This study explores the impact of technological innovation on CEE from two dimensions: technological innovation resources (TIR) and technological innovation capacity (TIC), which can be measured by the proportion of government technology expenditure in total expenditure and patent applications, respectively.

(2) Urbanization level (URL): In existing studies, the ratio of the urban population to the total regional population is widely used to characterize the level of urbanization [51,52,53]. With the growth of the urban population, a large amount of public infrastructure is needed to meet increasing resource demands, which increase urban energy consumption and carbon emissions. Some scholars take a positive view on urbanization [54], holding that the impact of urbanization on CEE presents a U-curve relation. The improvement of urbanization can implement energy use efficiency and further restrains carbon emissions and improves eco-environmental quality [55]. The population urbanization rate was selected to represent the extent of urbanization in this study.

(3) Industrial structure (IS) is an essential influencing component of CEE [56]. As a pillar industry for the national economy, the secondary industry based on the energy-intensive industry promotes carbon emissions, thereby reducing CEE. The industrial structure is represented by the secondary industry output value ratio to GDP.

(4) Economic development level (EDL): Economic growth directly affects carbon emissions [57]. Economic development generates a continuous increase in carbon emissions through resource consumption while leading to energy-saving with the advancement of the development process [58]. Developing countries and regions still have a relatively high demand for resources and energy, implying that economic development is negatively related to CEE. The level of economic development is measured by the GDP per capita.

(5) Foreign trade (FT): Foreign trade has been a crucial factor influencing carbon emissions. Previous studies indicated that environmental pollution transferred from developed countries to developing countries through foreign direct investment (FDI) forms a “pollution refuge” and accelerates the growth of carbon emissions [59]. On the other hand, importing some high-energy products instead of regional production can reduce energy consumption and improve the CEE. FDI represents foreign trade.

### 3.5. Panel Data Models

This empirical analysis used panel data models to further explore the interaction between technological innovation and CEE. All the variables discussed in this study are in natural logarithms. The panel data model was established as follows [60,61]:

(17) $$lnCEE_{it}=\alpha_{0}+\beta_{1}lnTIR_{it}+\theta_{1}lnURL_{it}+\theta_{2}lnIS_{it}+\theta_{3}lnEDL_{it}+\theta_{4}lnFT_{it}+\mu_{i}+\varepsilon_{it}$$

(18) $$lnCEE_{it}=\alpha_{0}+\beta_{2}lnTIC_{it}+\theta_{1}lnURL_{it}+\theta_{2}lnIS_{it}+\theta_{3}lnEDL_{it}+\theta_{4}lnFT_{it}+\mu_{i}+\varepsilon_{it}$$

where $\beta_{k}$ and $\theta_{m}$ are the coefficients of kernel explanatory variables and control variables, respectively. $\mu_{i}$ can be interpreted as the random heterogeneity specific to the *i*-th observation and is constant over time. $\varepsilon_{it}$ represents the random error term, explaining other uncaptured variables in the model that affect carbon emissions’ efficiency. $\alpha_{0}$ is a constant.

### 3.6. Data Sources

The panel data of 48 cities in 3 urban agglomerations were collected for empirical analysis from 2003 to 2017. All the data in this study were retrieved from official open data. The carbon emissions data of China’s cities were obtained from China Emission Accounts and Datasets (CEADs) (https://www.ceads.net/ (accessed on 12 November 2020)). Fixed assets investment, the number of employees, electricity consumption, GDP, the proportion of government technology expenditure in total expenditure, patent applications, population urbanization rate, the ratio of secondary industry output value to GDP, GDP per capita, and FDI were extracted and measured from the China city statistical yearbook (2004–2018), statistical yearbooks from each city (2004–2018). The descriptive statistics of the driving factors’ data are shown in Table 3.

## 4. Results

### 4.1. Spatiotemporal Distribution Pattern Analysis of CEE

**Figure 2:**
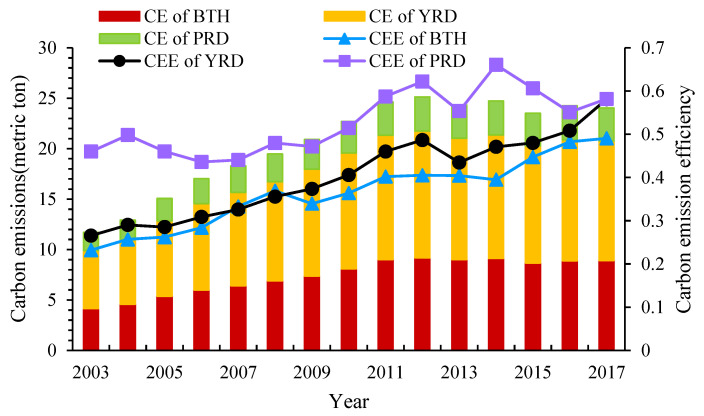
Evolution of China’s carbon emissions (CE) and CEE in the three major urban agglomerations from 2003 to 2017.

Based on the super-SBM model considering the undesirable output, we estimated the CEE of 48 cities in the three major urban agglomerations. According to previous studies [62,63], the average value is used to represent the overall level of CEE in a region. The temporal trends of CEE and carbon emissions (CE) of the three major urban agglomerations in China are described in Figure 2. In general, the CEE of the three urban agglomerations showed a slow growing trend during the studied period, hinting at a trend of significant carbon emissions reduction and energy-saving. Nevertheless, increases of CEE in each urban agglomeration differed, with PRD fluctuating more than others over time. From 2003 to 2017, the total value of the average CEE of BTH, YRD, and PRD were 0.2585, 0.3156, and 0.1218, respectively. Except for the average CEE in 2007 and 2008, which were highest in PRD, followed in order by BTH and PRD, CEE of the three major urban agglomerations followed the pattern of “PRD > YRD > BTH”. Overall, PRD played a greater role in sustainable economic development and energy improvement than BTH and YRD. The total carbon emissions of the three major urban agglomerations showed a year-to-year growth until 2012, when they peaked at 25.1271 metric tons. After that, the emissions reversed the increasing trend by setting and adjusting reduction targets at five-year intervals. During the study period, carbon emissions followed the pattern of “YRD > BTH > PRD”, indicating that YRD was still the largest contributor to carbon emissions in the three major urban agglomerations. Totally, relying on its geographical proximity to Hong Kong and Macao and the advantages of previous national policies, PRD has strong capabilities of innovation research and development with rapid economic growth, resulting in inputting capital and energy that can increase desired outputs to a greater extent and reduce undesired outputs. YRD has relatively high carbon emissions due to a large number of cities and massive industrial agglomeration. However, benefiting from its profound economic basis, the CEE is steadily improving in YRD. The energy consumption structure of BTH is mainly dominated by fossil energy and the contradiction between economic development and environmental pollution is most extruded in the three main agglomerations. With the practice of low-carbon development in recent years, the CEE in BTH has been effectively improved, while the green development patterns still need to be explored.

**Figure 3:**
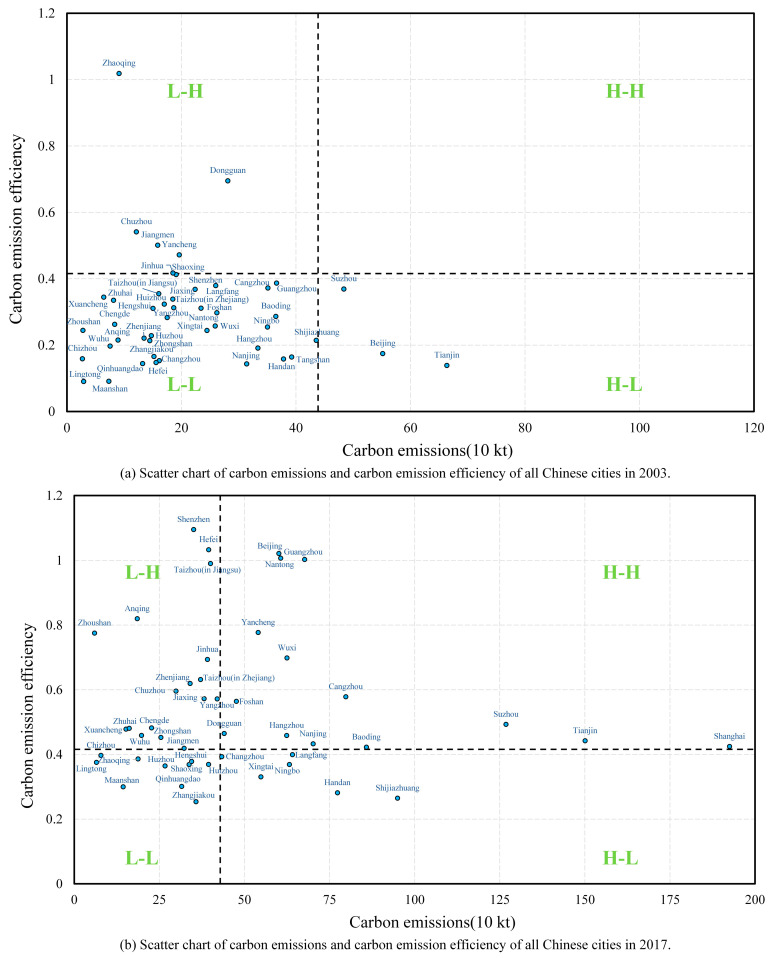
Mechanism of residents’ waste classification behavior.

To further analyze the changes in carbon emissions and CEE of each city within the three major urban agglomerations, we established a four-quadrant scatter plot with carbon emissions as the abscissa and CEE as the ordinate for two years, 2003 and 2017 (Figure 3). Taking the average value of carbon emissions and CEE of the total sample as the origin coordinate (42.8679, 0.4157), the quadrants corresponded to: “high-emission, high-efficiency” (H-H), “low-emission, high-efficiency” (L-H), “low-emission, low-efficiency” (L-L), and “high-emission, low-efficiency” (H-L). Most cities shifted from L-L in 2003 to L-H and H-H in 2017, which reflected a decline in the number of cities with lower CEE and a transition to cities with higher CEE. Specifically, in 2003, the number of H-L, L-L, L-H, and H-H cities were 5, 37, 6, and 0, respectively, with the vast majority of the cities in the three major urban agglomerations being of type L-L. Cities in the three major urban agglomerations underwent tremendous changes over time. In 2017, the number of H-L, L-L, L-H, and H-H cities were 6, 10, 17, and 15, respectively, where H-H cities accounted for the majority in BTH, and L-H cities accounted for the majority in YRD and PRD. Overall, the transformation of BTH underwent a decline in CEE, gathering to a higher CEE with the increase in carbon emissions over time. YRD and PRD shifted from low efficiency to high efficiency while paying more attention to controlling the growth of carbon emissions.

**Figure 4:**
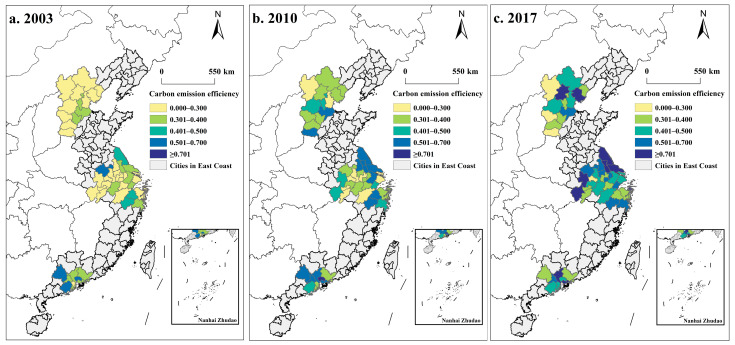
Spatial distribution of CEE in the three major urban agglomerations of China.

Three years (2003, 2010, 2017) were selected to map the spatial distribution of CEE in the three major urban agglomerations using ArcGIS 10.8 software (Figure 4). Total CEE in the three major urban agglomerations shows an upward trend. While in 2003 there were 28 cities with an efficiency of less than 0.3, there were only 4 in 2017. Moreover, the spatiotemporal patterns of CEE had different evolution characteristics. In BTH, low-efficiency cities formed a C-structure surrounding Langfang, Cangzhou, and Hengshui, which had relatively high efficiencies in 2003. In 2010, CEE was significantly enhanced, and the original higher-efficiency cities expanded into an I-shape along the north–south direction. In 2017, the higher-efficiency cities in BTH were concentrated in the northeast and developed a high-efficiency city cluster with the dual-core of “Beijing-Tangshan”. Since the economic development of the BTH region mainly radiates from Beijing and Tianjin to surrounding cities, the Beijing-Tianjin region benefits from the concentration of talents, capital, and technology, which can more effectively promote the transformation of energy-intensive industries into green and low-carbon industries. However, other regions are constrained by economic factors, which fail to achieve rapid industrial transformation, resulting in significant differences in the spatial distribution of CEE in BTH. The higher CEE cities in YRD were distributed with a scattered pattern and the high-value cluster in Yancheng, Taizhou, and Nantong formed a cluster in blocks from 2003 to 2017. This shows that the overall improvement of CEE was significant during this period and gradually spread to southwestern cities. After more than ten years of economic development, especially with the promotion of the integrated development of the Yangtze River Delta region as a national strategy, the quality of low-carbon development in YRD has been continuously improved. The relative value of CEE in PRD changed from “high at the edge and low at the middle” in 2003 to “high at the middle and low at the edge” in 2017, and the average CEE was at a high level. In general, the ecology and economy of each city in PRD can develop simultaneously due to the positive role played by the central cities, such as Guangzhou and Shenzhen. From an evolution perspective, the efficiency of the three major urban agglomerations presented a spatial pattern of “PRD > YRD > BTH”, which is consistent with the above results of spatiotemporal distribution. This indicated that the difference in CEE between the cities was still the focus of the development of the three major urban agglomerations. At the same time, it was also indispensable to seize the opportunity for the timing of the collaborative governance of BTH.

### 4.2. Spatiotemporal Difference Analysis of CEE

To further reveal the differences in CEE distribution, the Dagum Gini coefficient was used to calculate and decompose CEE disparity in each of the three major urban agglomerations from 2003 to 2017. The total Gini coefficient ranged from 0.1745 to 0.2718 and decreased from 0.2718 in 2003 to 0.2334 in 2017, with a drop of 14.13%. It derives from the fact that the Chinese government advocates putting energy conservation, emission reduction, and green development ahead of economic growth in recent years, thereby optimizing the ecological environment and alleviating the uncoordinated development among cities. Specifically, the total difference in CEE had a fluctuating and downward trend during the study period, which implies that the differences of CEE within cities became smaller. The overall Gini coefficient showed a strong reduction of 26.30% from 2004 to 2006. Subsequently, the overall Gini coefficient fluctuated between 0.1745 and 0.2086 from 2006 to 2016, while this unbalanced status started to collapse and increased to 0.2334 in 2017.

**Figure 5:**
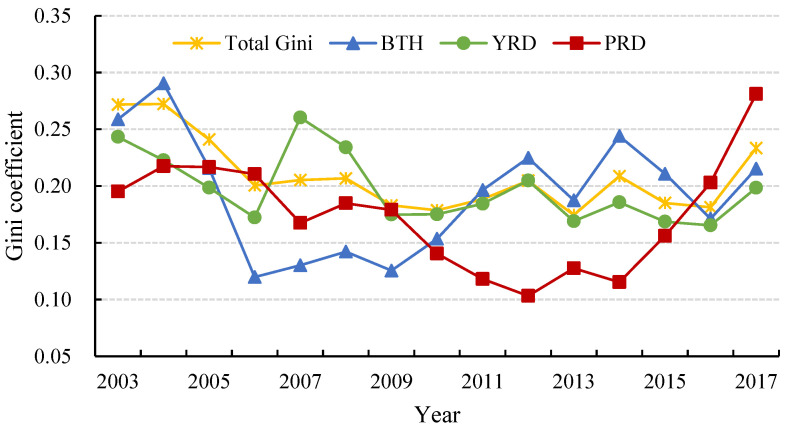
Total and intra-regional Gini coefficient of CEE in China’s three major urban agglomerations from 2003 to 2017.

Figure 5 demonstrates the variation of the total and intra-regional Gini coefficient of CEE from 2003 to 2017. The Gini coefficient within urban agglomerations increased in PRD but decreased in BTH and YRD. From the perspective of its evolution, the difference within PRD showed first a downward trend and then an upward trend during the study period, with 2014 as the turning point with the coefficient of 0.1153. The gap in YRD changed significantly and its evolution process can be divided into three stages: the first stage was from 2003 to 2006, when the intra-regional Gini coefficient presented an approximately linear decrease with a slope of −0.0237, the second stage experienced an inverted V-shaped polyline from 2006 to 2009, and the third stage was from 2009 to 2017, when the intra-regional Gini coefficient fluctuated steadily, ranging from 0.1654 to 0.2048. The 12th Five-Year Plan attaches great importance to the development of the ocean and economic development, thereby promoting the ecological and economic improvement of YRD in coastal areas. Under the guidance of the coordinated development strategy of urban agglomerations, the upgrading of industrial structure in BTH has driven the emergence and development of low-carbon industries and narrowed the gap among cities. Setting 2003 as the base period, PRD was the only urban agglomeration with an increasing internal gap compared to 2017. The intra-regional Gini coefficient fluctuated and declined until 2014, followed by a sharp increase to 0.2810 in 2017, which indicated a serious expansion in the unequal distribution of CEE. According to the analysis of the spatiotemporal distribution pattern above, although the average CEE of PRD is at a high level, the internal development is extremely uneven. The advantages of central cities in geographical location, resource endowment, and economic quality make them farther apart from low-level development cities such as Zhongshan and Zhaoqing. This single-center urban agglomeration structure eventually leads to the irrational allocation of regional resources, thus hindering the low-carbon development of each city in PRD [64]. As a result, the hierarchical differentiation of city development has become increasingly solidified, which has led to the imbalance of low-carbon development among cities in recent years.

**Figure 6:**
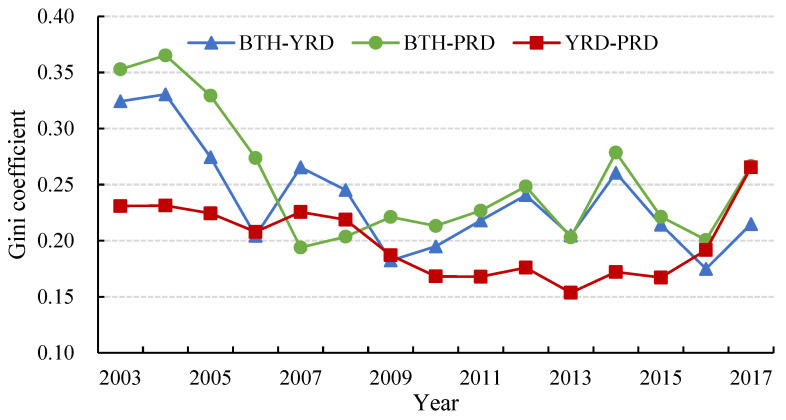
Inter-regional Gini coefficients of CEE in China’s three major urban agglomerations. From 2003 to 2017.

Figure 6 depicts the gap between the urban agglomerations and the variation trend of CEE. The inter-regional Gini coefficient evolution trends for BTH-YRD and BTH-PRD indicate a fluctuating downward trend, while the inter-regional Gini coefficient for YRD-PRD shows a gentle downward trend until 2015, followed by a drastic rise between 2015 and 2017. The differences between the maximum and minimum of BTH-YRD, BTH-PRD, and YRD-PRD are 0.1555, 0.1589, and 0.1117, respectively. The results show that the gap of CEE was gradually narrowed between BTH and the other two urban agglomerations, while the gap between YRD and PRD presented a falling–rising evolution until 2017 when it surpassed the gap in 2003. Since the coordinated development of urban agglomerations has become an important national development strategy, there is still a scope for improving CEE for many cities.

**Figure 7:**
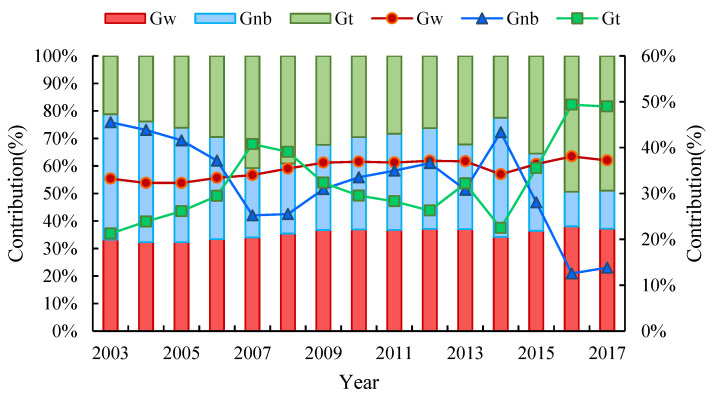
The sources of the differences in CEE and the trends of their contributions.

The sources of the differences in CEE and the trends of their contributions are presented in Figure 7. During the study period, the contribution rate of the inter-regional net differences generally showed a downward trend, while the contribution rate of the intensity of transvariation was the opposite, showing an upward trend of fluctuation. The contribution rate of the intra-regional differences did not change significantly. From the dynamic evolution processes, the contribution rate of the intra-regional differences was relatively stable, between 32.30% and 38.07%, and exhibited an overall increasing trend. From 2003 to 2017, the contribution rate of the inter-regional net differences and the intensity of transvariation underwent repeated increases and decreases. The contribution rate of the inter-regional net differences fluctuated more strongly. Specifically, we can identify three stages. From 2003 to 2007, the contribution of the inter-regional net differences showed a decreasing trend, reaching 25.23% in 2007. The second stage was from 2007 to 2014, which experienced repeated fluctuations. The contribution of the inter-regional net differences increased from 25.23% in 2007 to 62.85% in 2012, and then decreased to 36.61% in 2013, finally reaching its highest rate of 43.34% in 2014. From 2014 onwards, the contribution of the inter-regional net differences showed a sharp decline in 2016, when it reached the lowest value of 12.57%, after which it slightly increased in 2017. The variation of the intensity of transvariation was roughly opposite to the contribution rate of the inter-regional net differences. The intensity of transvariation reveals the impact of cross-term statistics among the three major urban agglomerations on the overall CEE difference. The contribution rate of the intensity of transvariation showed an increasing trend until 2007 and decreased to 26.27% in 2012. Subsequently, the rate experienced a falling–rising–falling evolution and reached the highest of three contribution rates in 2016 and continued to 2017, indicating that inter-regional and intra-regional interactions gradually increased.

### 4.3. Impact of Technological Innovation on CEE in the Three Major Urban Agglomerations

Before performing the panel data regression, it is necessary to test whether the core explanatory variables lnTIR and lnTIC and control variables lnURL, lnIS, lnENL, and lnFT were stationary during 2003–2017. Specifically, we adopted two types of panel unit root tests: the LLC test [65] and the Fisher-ADF test [66]. The results all rejected the unit root null hypothesis at the 1% significance level, indicating that the test data were stationary without a unit root, which avoids the possibility of spurious regression (Table 4).

To determine the optimal regression model suitable for comprehensively evaluating the impact of technological innovation on CEE, two steps are required to test whether the random effect model and the fixed effect model need to be used. The Lagrange multiplier (LM) test was first calculated to decide the random effect. The rejection of the null hypothesis indicated that the panel effect existed between cities, suggesting that the random effect model was appropriate. Subsequently, the Hausman test was performed to verify whether the fixed effect was superior to the random effect. A rejection of the null hypothesis indicates that the fixed effect model should be adopted for regression estimation. Due to the diverse impacts of different indicators representing technological innovation on CEE, two core explanatory variables were separated and regressed with CEE to explore the influence. Both results of the LM test significantly rejected the null hypothesis at the 1% level and the random effect model was accepted. Similarly, the Hausman test values of 21.94 and 32.39 (both *p* < 0.000) revealed the evidence to embrace the fixed effect model (Table 5).

The results indicate that both TIR and TIC exerted significant positive effects on CEE at the 1% significance level with coefficients of 0.0806 and 0.153, respectively. The indicators of technological innovation were found to profoundly affect CEE in the three urban agglomerations. This confirms that the government can scale up the input funding in technological innovation to promote CEE. Additionally, new technologies can be directly applied to develop clean energy and establish clean production modes that reduce carbon emissions. Furthermore, among the technological innovation indicators, patent applications seem to have the largest impact on CEE, of which the estimated coefficient is comparatively higher than the proportion of government technology expenditure in total expenditure. This indicates that the promotion of new technological applications has a large effect on CEE. Every 1% increase in patent applications would enhance CEE by 0.153%. Patent applications can effectively characterize the level of knowledge output in technological innovation and evaluate the innovation capacity of a region. Patents play a unique role in developing innovative products in specific sectors, including high carbon emission sectors as well. They act as a bridge for gathering huge investments and R&D research from governments, companies, and private organizations to create green technologies for fossil energy-saving. Meanwhile, every 1% increase in the proportion of total government technology expenditure causes CEE to increase by 0.0806%. Investments in technology are necessary to advance the energy transition from fossil fuel to clean energy sources and optimize the industrial structure by combining advanced elements with environmental protection. As one of the major sources of investment in technological innovation, government expenditure integrates government guidance and economic market operations to attract companies to develop low-carbon and high-efficiency technology equipment, reducing the consumption of fossil energy resources directly and indirectly.

Regarding control variables, the level of economic development had a significant positive effect in Model 3 and Model 4, with coefficients of 0.224 and 0.114 at the 1% and 5% significance levels, respectively. The three major urban agglomerations are the economically developed regions with the largest economies, which is conducive to improving emission efficiency by attracting capital and high-tech talents. Moreover, economic growth triggers technological change and economic structure evolution, thereby curbing the increase in carbon emissions in this stage. Urbanization and industrial structure variables have a significant negative effect on CEE at the 1% level. In the process of urbanization, rapid population concentration and industrial activities accelerate energy consumption to meet the need for massive infrastructure and energy-intensive products. Meanwhile, it can be observed that FDI is negative at the 10% level in Model 4, suggesting that every 1% increase in per capita FDI caused CEE to reduce by 0.0459%. However, the coefficient is not significant in Model 3.

### 4.4. Heterogeneity Analysis of the Impact of Technological Innovation on CEE

To further analyze the heterogeneous influence of technological innovation, we estimated the impact of TIR and TIC in BTH, YRD, and PRD. The results of panel data regression are reported in Table 6.

The results of the Hausman test support the fixed effect model in BTH and the random effect model in YRD and PRD. The TIR coefficients were 0.107, 0.165, and 0.0739, significantly positive at the 10%, 1%, and 10% levels, showing that the government’s technology expenditure as a proportion of total expenditure improved CEE in BTH, YRD, and PRD by various degrees. The effect was greatest in YRD. Every 1% increase in the proportion caused CEE in YRD to increase by 0.165%. YRD, including Shanghai, Nanjing, Suzhou, and Hangzhou, is located in one of the most economically developed zones, with the highest degree of openness and the strongest innovation potential on the eastern coast of China. It is at an important intersection between the “Belt and Road” and the Yangtze River Economic Belt, with a pivotal strategic position in driving China’s high-quality and regional coordinated development. Due to the higher similarity of industries in YRD with low resource allocation efficiency and fierce competition in the region, the government expands science and technology financing to achieve innovation breakthroughs, thereby promoting industrial coordination. The emergence of new technologies creates an environment that promotes productivity as well as the opportunity for energy conservation and carbon emission reduction. The influence coefficient of TIR in the BHT is second only to that in YRD, where every 1% increase would cause CEE to increase by 0.107%. As the political center of China, the central government works in close collaboration with local governments to guide the coordinated development of BTH. The high concentration of heavy industry and utilization of coal-based energy have long made BTH one of China’s most polluted urban agglomerations, which attracts great attention from the government. The proposed target of peak carbon dioxide emissions and carbon neutrality promotes the flow of the government’s technological investment to high-tech and cultural innovation industries, strongly impacting CEE. The impact coefficient of PRD is lowest in the three major urban agglomerations, indicating that every 1% increase causes CEE to increase by 0.0739%. This may be because pollution in PRD is not considered the most urgent issue in the process of economic development yet. Therefore, the government’s financing of technology is preferably used to improve the market competitiveness of products and promote the upgrading of traditional manufacturing industries.

Referring to impact analysis of TIC in each urban agglomeration, the results of the Hausman test support the fixed effect model in BTH and YRD and the random effect model in PRD. The coefficients of TIC are all significantly positive at the 1% level in the three urban agglomerations, with the rank of “BTH > YRD > PRD”. Compared with PRD, there is a small difference in the coefficients of BTH and YRD. Every 1% increase in the number of patent applications would improve CEE in BTH and YRD by 0.25% and 0.23%, respectively. BTH and YRD are home to many top universities and research institutes, with strong knowledge spillover effects and remarkable innovation achievements. High innovation capability plays an important role in adjusting industrial structure and improving the utilization of energy efficiency, which is conducive to building a low-carbon energy consumption system. Although TIC exerts a positive impact on CEE of PRD, the coefficient is only 0.132, indicating that a point increase in patent applications causes CEE to grow by 0.132%. The emissions control by the improvement of innovation capability may shift to focus on the growth and productivity of the economy, thereby reducing the impact of innovation output on emissions reductions. In summary, technological innovation contributes positively to the improvement of CEE. Still, its influence is affected differently by factors such as resource advantages, government policies, and the opening-up level of different urban agglomerations.

## 5. Conclusions

Technological innovation is the driving force for establishing a low-carbon economic system, thereby promoting an overall green transformation of economic and social development. To explore how technological innovation affects CEE, the super-SBM approach combined with undesired outputs was applied to estimate the CEE of 48 cities in China’s three major urban agglomerations from 2003 to 2017. The Dagum Gini coefficient was used to analyze CEE’s distribution difference. Moreover, we applied the panel regression model to analyze the effects of technological innovation on CEE. Given the large efficiency differences of the three urban agglomerations, we regressed the influencing factors of BTH, YRD, and PRD to explore the heterogeneity of technological innovation. The conclusions were obtained as follows.

First, from 2003 to 2017, the overall CEE increased steadily and the growth in carbon emissions shifted from rapid to gradual in the three major urban agglomerations, moving from the “low-emission, low-efficiency” areas to the “low-emission, high-efficiency” and “high-emission, high-efficiency” areas. Specifically, the CEE of PRD was the highest, followed by that of YRD, and that of PRD was the lowest, except in some years. The spatial distribution pattern of “PRD > YRD > PRD” showed that high-value efficiency cities were largely concentrated in YRD and PRD within the study period. The CEE of BTH gradually improved, while its overall efficiency was still relatively low.

Second, the development of CEE was spatially heterogeneous in the three major urban agglomerations. The total Dagum Gini coefficient indicated that the gap of CEE in the three major urban agglomerations fluctuated less over time. From 2003 to 2017, the decomposition of the Dagum Gini coefficient showed that the PRD gap increased while BTH and YRD decreased. Driven by the coordinated development of BTH, the gap between BTH and the other two urban agglomerations became smaller. However, there was still a large gap among cities. Besides, the main source of urban agglomeration difference in CEE shifted from inter-regional net differences to intensity of transvariation. The contribution of the intra-regional differences was relatively stable during the study period.

Third, the impact of technological innovation was found to have a significantly positive effect on CEE in these three urban agglomerations. Specifically, the influence coefficient of the proportion of government technology expenditure as a function of total expenditure (0.124) was lower than that of the number of patent applications (0.0806). Moreover, different control variables had different effects on CEE. Industrial structure, urbanization level, and foreign trade had an inhibitory effect on CEE, while the economic development level significantly improved CEE.

Fourth, the influence of technological innovation is heterogeneous in the three major urban agglomerations. In general, technological innovation is found to significantly affect CEE in each urban agglomeration. In terms of technological innovation resources, the influence coefficients of the proportion of government technology expenditure in total expenditure presented the order of “YRD > BTH > PRD”. The influence coefficient of patent applications in BTH is the highest, followed by YRD, and PRD is the lowest. Influenced by the characteristics and scale of urban agglomerations, there are significant differences in the degree of impact of technological innovation on CEE.

This study responds to the long-standing controversy over the impact of technological innovation on CEE and confirms that technological innovation is generally beneficial for reducing urban carbon emissions and improving productivity. It provides a reference for predicting the effect of technological innovation and formulating government policy. Notably, the research framework of this study can be applied to other regions and extended to explore other influencing factors on environmental pollution.

## 6. Policy Suggestions

In view of the differences in CEE of the three major urban agglomerations, policies and measures should be formulated according to local conditions. Based on the above empirical findings and discussions, we make the following suggestions for policymakers.

First, government financial support for technological innovation should be increased. On the one hand, the government should compensate for enterprises’ insufficient funds by expanding technical financial expenditures, directly promoting enterprises to actively create clean products, and adjusting internal unreasonable structures. On the other hand, the government should make full use of innovative financial investment to facilitate innovative talents and social capital flowing into the economic market, thereby accelerating the research on low-carbon and decarbonization technology development. Moreover, the government should also strengthen cooperation with social institutions to form a low-carbon technology investment and financing system.

Second, the transformation of green and low-carbon technologies should be promoted to inject strong technological momentum into green development. It is necessary to give full play to the positive role of the market in the direction and path selection of technology research, thereby enhancing the effective connection of low-carbon technologies, innovation capital, and high-efficiency industries. The government should reinforce the introduction and implementation of technological innovation protection policies. It should also build a platform for optimal allocation and information sharing of technological research and development resources to accelerate the transformation of technological innovation achievements into real productivity.

Third, the construction and development of the three major urban agglomerations of China should be coordinated. BTH, YRH, and PRD should establish low-carbon pilot demonstration areas in their key cities and replicate these experiences. Based on its characteristics, each urban agglomeration should formulate appropriate strategies to strengthen the low-carbon emission reduction mechanism. Specifically, cities in YRD should promote the free exchange of innovation elements between regions and form a sound inter-regional innovation network, which is conducive to knowledge spillover, technology progress, and the generation of new technologies. Cities in BTH should adopt coordinated emission reductions of urban agglomerations rather than individual emission reduction plans. The government should propel the emergence of a coordinated carbon emission reduction mechanism for BTH by formulating regulatory policies. PRD should promote regional integrated development and maintain the effect of coordinated emission reduction. On this basis, industry-driven technological innovation should continue to be strengthened to support regional carbon emission reduction and high-quality economic development.

## Figures and Tables

**Table 1:** Socio-demographic profile of the respondents.

Variables	Primary Indicators	Secondary Indicators	Units
Input	Capital investment	Fixed capital stock	10^8^ yuan
	Labor input	Number of employees	10^4^ people
	Energy input	Electricity consumption	10^4^ KWH
Output	Desirable output	GDP	10^8^ yuan
	Undesirable output	CO_2_ emissions	10 kt

**Table 2:** The indicator system of influencing factors on CEE.

Type	Primary Indicators	Secondary Indicators	Units
Explained variable	Carbon emission efficiency	-	-
Core explanatory variable (Technological innovation level)	Technological innovation resources	The proportion of government technology expenditure in total expenditure	%
	Technological innovation capacity	Patent applications	PCS
Control variates	Urbanization level	Population urbanization rate	%
	Industrial structure	The ratio of secondary industry output value to GDP	%
	Economic development level	GDP per capita	Yuan
	Foreign trade	Foreign direct investment	10^4^ USD

**Table 3:** Descriptive statistics of driving variables of CEE.

Region	Variable	Symbol	Mean	St. Dev.	Min	Max
Total	Carbon emission efficiency	CEE	0.4161	0.1868	0.0904	1.2303
	Technology innovation resources	TIR	2.36	2.06	0.04	12.65
	Technology innovation capacity	TIC	12,910	20,986	17	161,619
	Urbanization level	URL	58.41	17.79	16.05	100
	Industrial structure	IS	50.46	8.58	19.01	74.73
	Economic development level	EDL	53,189	35,262	4876	199,017
	Foreign trade	FT	1,190,808	295,610	1110	2,433,000
BTH	Carbon emission efficiency	CEE	0.365	0.167	0.139	1.23
	Technology innovation resources	TIR	1.163	1.327	0.118	6.584
	Technology innovation capacity	TIC	5911.282	15,239.714	69	99,167
	Urbanization level	URL	50.438	15.008	28.17	86.5
	Industrial structure	IS	47.998	9.056	19.01	60.08
	Economic development level	EDL	35,922.908	25,482.697	6555	128,994
	Foreign trade	FT	173,566.26	384,191.55	1110	2,433,000
YRD	Carbon emission efficiency	CEE	0.402	0.167	0.09	1.033
	Technology innovation resources	TIR	2.691	2.041	0.045	12.648
	Technology innovation capacity	TIC	14,728.321	20,870.649	17	121,496
	Urbanization level	URL	56.549	14.344	16.05	89.6
	Industrial structure	IS	51.643	7.88	29.83	74.73
	Economic development level	EDL	56,403.364	35,291.689	4876	199,017
	Foreign trade	FT	194,105.36	280,460.02	1338	1,851,378
PRD	Carbon emission efficiency	CEE	0.528	0.22	0.214	1.185
	Technology innovation resources	TIR	3.139	2.275	0.167	9.686
	Technology innovation capacity	TIC	17,765.267	25,646.189	212	161,619
	Urbanization level	URL	75.3	19.632	26.78	100
	Industrial structure	IS	50.607	9.099	23.48	64.33
	Economic development level	EDL	68,845	37,483.959	11,907	189,993
	Foreign trade	FT	206,185.91	161,679.63	18,135	740,126

**Table 4:** Results of panel unit root tests.

Variable	LLC Test		ADF-Fisher Test		Result
	Stat.	*p*-Value	Stat.	*p*-Value	
*lnTIR*	−7.8394	0.0000	16.7540	0.0000	Stationary
*lnTIC*	−4.7003	0.0000	2.4501	0.0071	Stationary
*lnURL*	−40.8640	0.0000	8.4049	0.0000	Stationary
*lnIS*	−5.5402	0.0000	3.4236	0.0003	Stationary
*lnENL*	−8.5315	0.0000	9.2944	0.0000	Stationary
*lnFT*	−7.9233	0.0000	5.0507	0.0000	Stationary

**Table 5:** Panel regression results for the three major urban agglomerations.

Variable	Random Effect		Fixed Effect	
	Model 1	Model 2	Model 3	Model 4
*lnTIR*	0.124 ***		0.0806 ***	
	(0.0192)		(0.0201)	
*lnTIC*		0.158 ***		0.153 ***
		(0.0176)		(0.0172)
*lnURL*	−0.426 ***	−0.428 ***	−0.545 ***	−0.559 ***
	(0.0756)	(0.0737)	(0.0776)	(0.0738)
*lnIS*	−0.239 ***	−0.187 ***	−0.239 ***	−0.262 ***
	(0.0720)	(0.0705)	(0.0715)	(0.0689)
*lnEDL*	0.208 ***	0.139 **	0.224 ***	0.114 **
	(0.0440)	(0.0437)	(0.0446)	(0.0430)
*lnFT*	0.0144	−0.0696 ***	0.0091	−0.0459 *
	(0.0152)	(0.0174)	(0.0164)	(0.0182)
*_cons*	−0.717	−0.502	−0.251	0.347
	(0.423)	(0.394)	(0.424)	(0.388)
*R^2^*	0.1949	0.2013	0.1908	0.1790
*F-Statistics*			195.38	193.64
*Hausman test (Prob.)*	21.94 (0.0000)	32.39 (0.0000)		

Note: ***, **, and * denote significance levels of 1%, 5%, and 10%, respectively.

**Table 6:** Panel regression results of TIR and TIC in each urban agglomeration.

Variable	BTH				YRD				PRD			
	Random Effect		Fixed Effect		Random Effect		Fixed Effect		Random Effect		Fixed Effect	
*lnTIR*	0.116 *		0.107 *		0.165 ***		0.128 ***		0.0739 *		−0.0146	
	(0.0467)		(0.0484)		(0.0231)		(0.0286)		(0.0322)		(0.0450)	
*lnTIC*		0.236 ***		0.250 ***		0.243 ***		0.230 ***		0.132 ***		0.122 **
		(0.0431)		(0.0492)		(0.0210)		(0.0223)		(0.0347)		(0.0417)
*Control*	Yes	Yes	Yes	Yes	Yes	Yes	Yes	Yes	Yes	Yes	Yes	Yes
*_cons*	−1.909	−2.931 ***	−1.120	−2.663 **	0.833	0.627	0.408	0.506	−0.649	−0.847	−0.964 ***	−0.914
	(0.990)	(0.832)	(1.032)	(0.862)	(0.594)	(0.508)	(0.638)	(0.513)	(0.766)	(0.891)	(0.822)	(0.971)
*R* ^2^	0.3498	0.4205	0.3195	0.4015	0.2979	0.4100	0.2861	0.4003	0.5000	0.5322	0.4622	0.5314
*F-Statistics*			12.59	17.61			21.36	42.77			22.72	26.22
*Hausman test (Prob.)*	25.89 (0.0001)	23.72 (0.0002)			8.29 (0.1411)				8.79 (0.1176)	0.68 (0.9840)		

Note: ***, **, and * denote significance levels of 1%, 5%, and 10%, respectively.

## Data Availability

The data that support the findings of this study are available upon request from the corresponding author.
